# Dietary Compounds for Targeting Prostate Cancer

**DOI:** 10.3390/nu11102401

**Published:** 2019-10-08

**Authors:** Seungjin Noh, Eunseok Choi, Cho-Hyun Hwang, Ji Hoon Jung, Sung-Hoon Kim, Bonglee Kim

**Affiliations:** 1 College of Korean Medicine, Kyung Hee University, Seoul 02453, Korea; nohapril@khu.ac.kr (S.N.); eschoi1210@khu.ac.kr (E.C.); chohyun.hwang@khu.ac.kr (C.-H.H.); 2 Department of Pathology, College of Korean Medicine, Graduate School, Kyung Hee University, Seoul 02453, Korea; johnsperfume@khu.ac.kr (J.H.J.); sungkim7@khu.ac.kr (S.-H.K.)

**Keywords:** dietary compounds, natural compounds, prostate cancer, apoptosis, angiogenesis, metastasis, MiRNA, multi drug resistance

## Abstract

Prostate cancer is the third most common cancer worldwide, and the burden of the disease is increased. Although several chemotherapies have been used, concerns about the side effects have been raised, and development of alternative therapy is inevitable. The purpose of this study is to prove the efficacy of dietary substances as a source of anti-tumor drugs by identifying their carcinostatic activities in specific pathological mechanisms. According to numerous studies, dietary substances were effective through following five mechanisms; apoptosis, anti-angiogenesis, anti-metastasis, microRNA (miRNA) regulation, and anti-multi-drug-resistance (MDR). About seventy dietary substances showed the anti-prostate cancer activities. Most of the substances induced the apoptosis, especially acting on the mechanism of caspase and poly adenosine diphosphate ribose polymerase (PARP) cleavage. These findings support that dietary compounds have potential to be used as anticancer agents as both food supplements and direct clinical drugs.

## 1. Introduction 

Cancer is one of the major causes of mortality in the world and prostate cancer is ranked as the third most commonly diagnosed cancer in the world [1]. Chemotherapy is one of the commonly used therapy with surgery, radiation therapy, immunotherapy, and hormone therapy for treating prostate cancer. However, severe side effects of the drugs such as neutropenia, stomatitis, mucositis, diarrhea, and emesis have been widely reported [2]. The examples of cruel adverse-effects of chemotherapy can be found in docetaxel (taxotere) and cabazitaxel (jevtana), which are used for prostate cancer [3]. These two medications showed the edema, hypersensitivity, neurotoxicity, etc. in prostate cancer patients [4]. As a result, there are growing demands for development of new therapeutic drugs and prevention methods that have minimum damage with maximum effects against prostate cancer from various materials, including food. 

Food contains various compounds that regulate human physiological activities by mediating target signaling pathway [5]. These bioactive compounds, also called natural compounds, affect the incidence and prognosis of human diseases, including arthritis [6,7], Alzheimer’s diseases [8,9], and cancers [10,11,12,13,14]. A variety of compounds such as vitamin D, curcumin, lycopene, sulforaphane, epigallocatechin-3-gallate (EGCG), resveratrol, and piperine were reported to have potential anticancer effects [15]. Recent studies indicated that these compounds work as both supplements, which prevent the risks of cancer and direct anticancer drugs, which induce the apoptosis of cancer cells [16]. Curcumin, a representative example, was found to interact with miR-21/phosphatase and the tensin homolog (PTEN)/protein kinase B (Akt), thereby leading to apoptosis in human gastric cancer [17]. In addition to gastric cancer, curcumin has impressive effects in lung cancers, cervical cancers, prostate cancers, breast cancers, osteosarcoma, and liver cancers [18]. Resveratrol showed an anti-pancreatic cancer effect via regulation of microRNA (miRNA)-200 and sonic hedgehog (SHH) signaling pathway [19]. The skin, breast, prostate, lung, colon, and liver cancers were inhibited by resveratrol treatment [20]. In this way, considerable amounts of dietary compounds have the evidences of their efficacies on anti-carcinogenic actions. Thus, anti-prostate cancer effects of dietary compounds were reviewed and their mechanisms were discussed in this review.

Many mechanisms of anti-carcinogenesis were found from various studies; apoptosis, anti-angiogenesis, metastasis, drug-resistance, etc. [21]. Apoptosis is a programmed cell death, which is critical for removing unessential cells like tumor cells. Specific mechanisms of apoptosis in anti-carcinogenic action include poly (ADP-ribose) polymerase (PARP), caspase-3, 7, and 9, cellular FLICE (FADD-like IL-1β-converting enzyme)-inhibitory protein (c-FLIP). c-FLIP is a protein that decides the amount of apoptosis activity made in the body [22]. Anti-angiogenesis is an inhibition of novel blood vessel formation, thereby inducing nutrition deficiency of tumor cells [23]. Metastasis is transmission of tumor cells from the main tumor cells to other parts of the body. Well-known anti-metastasis mechanisms are the immune-stimulatory factors, including GM-CSF, IL-12; expression of anti-metastatic factors, including Kangai 1 (KAI1), NM23, and modified IgG Osteoprotegerin (OPG) [24]. Drug resistance is a mechanism that reduce the effective of drugs via enhanced DNA repair mechanism, aberrant expression of membrane transporters, adapted drug metabolism, suppression of apoptosis, increased cell survival mechanism, etc. [25]. Recently, many dietary compounds were found to take part in one or more mechanisms mentioned above, with low level of side effects [26]. 

In this review, effective dietary chemicals for treating prostate cancer were organized and discussed. In despite of many positive reports about dietary compounds as potent anti-prostate cancer drugs, there are some limitations such as difficulties in standardization, drug stability, and toxicity. Thus, in this review, the pros and cons of dietary compounds are discussed.

## 2. Results

### 2.1. Apoptosis and Dietary Compounds

Apoptosis, or programmed cell death, is one of the main target mechanisms for cancer therapy [22]. Numerous dietary compounds were found to regulate induce apoptosis. A total of thirty-nine compounds demonstrated the apoptotic effects upon prostate cancer cells or prostate cancer animal models, and most of the compounds were derived from plant organisms (Table 1). Auriculasin, originated from *Flemingia philippinensis* Merr. and Rolfe, have been used as a food ingredient as well as a source of medicine in China. This compound (5 μM) induced DNA fragmentation and chromatin condensation in lymph node carcinoma of the prostate (LNCaP) prostate cancer cells. Moreover, auriculasin elevated the expression of Bax, c-PARP, and reactive oxygen species (ROS), while repressed the Bcl-2 expression [27]. Chalcone cardamonin, derived from *Campomanesia adamantium* Myrtaceae is a native medicine used in Brazil. This compound increased the DNA fragmentation and decreased the nuclear factor kappa-light-chain-enhancer of activated B cells 1 (NF-κB1) in PC3 cells. These results supported the therapeutic potential of chalcone cardamonin for prostate cancer treatment [28]. Cyclohexenyl chalcones panduratin A and nicolaioidesin C were derived from *Boesenbergia pandurate* Roxb, which is mostly found in Southeast Asia. These compounds (5, 10, and 20 μM) increased the relative proportion of apoptotic cells between PC3 and LNCaP cells in a concentration-dependent manner. Additionally the effect of panduratin A was significantly higher than nicolaioidesin C in DU145 cells [29]. Delphinidin is a major anthocyanidin compound, which could be found in various plants such as pomegranates, berries, grapes, beets, and eggplants. Cytotoxic activity was reported in LNCaP cells (50, 100, and 150 μM), whereas not in DU145 or PC3 cells. In addition, the treatment of this dietary compound in LNCaP cells (50, 100, and 150 μM) exhibited the activation of c-caspase-3, -7, and c-PARP-1, c-PARP, c-histone deacetylase 3 (c-HDAC3), Bax, NADPH oxidase activator (Noxa), and the degradation of procaspase-8 and HDAC3. The decreased HDAC3 was also exhibited in delphinidin treated PC3 cells (50, 100, and 150 μM) [30]. Dimeric ellagitannins are newly found dietary compounds isolated from acetone extracts of *Cornus alba* Linne., which have been used as traditional herbal medicines for anti-inflammatory, hemostatic and diuretic purposes in Korea. Among the various compounds, cornusiin A, camptothin B, and an amorphous substance were reported to have antiproliferative and apoptotic effects when treated in DU145 (20, 50, and 100 μM), and LNCaP (5, 10, and20 μM) prostate cancer cells [31]. Docetaxel, originated from *Taxus baccata* L., is widely used as a therapeutic agent in prostate cancer treatment. This dietary substance is used for androgen dependent prostate cancer alone or in combination with conventional chemotherapy. In recent years, research showed it could even increase the survival rate of patients with early prostate cancer [32]. Docetaxel treated PC3 and DU145 cells demonstrated the activation of c-PARP, p-Bcl-2, Beclin1, and p-Janus kinase (JNK). Furthermore, it was reported that tea polyphenols (20 μM) or 3-methyladenine (10 μM) each combined with docetaxel (100 ng/mL) showed elevated expression of c-PARP when treated on PC3 and DU145 cells [33]. Emodin and rhapontigrnin were derived from *Rheum palmatum* Linne., a famous traditional Chinese drug conventionally used for hepatitis, gastritis, hypertension, and arteriosclerosis. It was reported that the two compounds (50 μM) could effectively induce apoptosis in DU145 cells [34]. FD1a, FD1c, FD1h, FD2a, FD2c, and FD2h are dietary compounds extracted from *Ficus deltoidea* Linne., a traditional Malaysian drug used for headaches, toothaches, sores, and wounds. When treated on PC3 and LNCaP cells, they induced the apoptotic efficacies by increased mitochondrial membrane potential (MMP) depolarization upon PC3 cells. It was demonstrated that the compounds (19 mg/mL) increased the levels of c-caspase-3, -7, and Bax against PC3 cells, while they (23 mg/mL) induced the activation of c-caspase-3 and -7 against LNCaP cells. [35]. Flavonoids were extracted from *Diospyros kaki* Linne., which is a persimmon leaf widely found in China and tropical and subtropical eastern Asia. It is reported that these compounds (12.5, 25 μg) up-regulated the expression of Bax, c-caspase 3 and down-regulated that of B-cell lymphoma 2 (Bcl-2) in PC3 cells [36]. Guttiferone F is a prenylated benzophenone derivative isolated from *Allanblackia stuhlmannii* Engl., increased the expression of c-caspase-9, -7, -3, and c-PARP both in PC3 and LNCaP cells [37]. Jungermannenone A and jungermannenone B originated from *Jungermannia fauriana* Beauverd activated the c-caspase-3 and c-PARP when exposed to PC3 cells [38]. Linalool and Linalyl acetate were extracted from *Lavandula angustifolia* Mill., a herbal medicine used in a form of oil extraction native to the western Mediterranean. Each of the compounds (2.5 µM) were examined the apoptotic effects against PC3, DU145 cells. Moreover, PC-3 xenograft model (10, 25, 50, 100, and 200 mg/kg) suppressed the tumor growth [39]. Matrine, sophocarpine, oxymatrine, sophocarpine, and xanthohumol are originated from *Sophorae flavescens* Aiton, which is a widely used as the complimentary medicine for various cancers in Traditional Chinese medicine. When experimented upon PC3 cells, the five compounds (0.1–2 mg/mL) showed clear apoptotic effect. Especially, matrine, and xanthohumol showed notable inhibitory effects against prostate cancer cells [40]. Nexrutine is a natural extract from *Phellodendron amurense* Rupr., which is used as an anti-inflammatory agent in traditional Chinese medicine. Nexrutine (10 μM) and docetaxel reduced the growth of PC3 cells, and it induced the c-caspase-3 and decreased the c-FLIP [41]. Ophiopogonin D, extracted from a traditional Chinese medicine *Ophiopogon japonicus* Linn. f., was shown to have apoptotic activity against PC3 and DU145 cells. Zongliang Lu et al. reported that this dietary compound (2.5, 5.0 μM) elevated the dose of Receptor-interacting serine/threonine-protein kinase 1 (RIPK1), Bcl-2-like protein 11 (Bim), while inhibiting the expression of c-RIPK1, caspase 8, c-caspase 8, caspase 10, c-caspase 10, and Bid [42]. Meanwhile, opiopogonin exerted significant tumor growth inhibition between PC3 and DU145 xenograft models in vivo at a dose of 2.5 or 5.0 mg/kg [42]. Panduratin A is a cyclohexenyl chalene derivative, isolated from an Indonesian and Thailand folk medicine, *Kaemferia pandurate* Roxb. Panduratin A (2.5, 5, 10, and 20 μM) showed the anti-proliferative effects upon androgen independent human prostate cancer cells, such as LNCaP, PC3, and DU145 cells through apoptosis induction and cell cycle regulation. Furthermore, apoptosis mediated by this dietary compound resulted in a decrease of Bcl-2, Bid, acinus and an increase in Bax, Fas-associated protein with death domain (FADD), TNF-related apoptosis inducing ligand (TRAIL), cleavages of PARP and caspase-3, -6, -8, and -9 [43]. Punicalagin is a dietary substance derived from *Punica granatum* Linne., which has been known to have anticancer activities in lung, breast, and cervical cancers. It was elucidated that the compound (100 μM) up-regulated the expression of c-caspase-3 and -8 in PC3 cells [44]. Physlain A and B are steroid compounds extracted from *Physalis alkekengi* Linne., a traditional Chinese herbal medicine, which is also used for medication of cancer. The compounds (5, 10, and 16 μM) suppressed the t-PARP and procaspase-3, and increased the c-PARP and c-caspase-3 [45]. Schizandrin (1) derivatives were extracted from *Schisandra grandiflora* Wall. The compounds (1 and 2 μM) induced the apoptotic effect in DU145 cells by cell cycle arrest, which eventually led to apoptotic results on prostate cancer cells [46]. Scutellarin is derived from *Scutellaria altissima* Linne., a common traditional Chinese medicine used for inflammatory diseases. Treatment of scutellarin (200, 400, and 600 μM) increased the cleavage of caspase-3, -9, and in PC3 cells [47]. Solanine is one of the main steroidal glycoalkaloids of *Solanum nigrum* Linne. Solamine (10–160 μM) was treated to DU145 cells and induced p38 pathway, p-ATF2, and Bax. However it resulted in a down-regulation of the expression level of Bcl-2. Furthermore, it (5 mg/kg) induced the apoptosis in DU145 inoculated six-to-eight-week-old male nude mice [48]. Terrestrosin D is extracted from *Tribulus terrestris* Linne., which is a medicinal plant distributed in the Mediterranean subtropical and desert climates. It has been used for treating urinary infections, inflammation, and cancer. This substance (2 and 5 μM) activated the apoptosis and increased VEGF secretion in PC3 cells. Moreover, it (25, 50 mg/kg) was discovered that terrestrosin D substantially suppressed tumor growth in the in vivo PC3 xenograft model [49]. Tetrandrine is a bisbenzylisoquinoline alkaloid purified from the root of *Stephania tetrandra* S. Moore. This compound (2.5, 5.0, and 10.0 μM) triggered the caspase-3 activity, expression of c-PARP and Bax, and suppressed the level of Akt and Bcl-2 in DU145 and PC3 prostate cells [50]. Thalicthuberine was isolated from *Hernandia albiflora* Kubitzki, an Australian endemic tree. This compound was treated to LNCaP (2.5 μM), CEM (5 and 10 μM), and VCR-R (60 μM), and the results showed an increase in caspase3/7 activity [51]. Triterpenoid plectranthoic acid is a dietary compound derived from *Ficus microcarpa* Linne. f., a traditional medicine in South Asia especially used for treating type-2 diabetes. When the compound was treated to DU145, PC3 and NB26 cells, up-regulation of Bax, c-PARP and c- caspase3, and down-regulation of Bcl-xl and Bcl-2 was exhibited [52]. Xanthohumol was derived from glands of strobiles of *Humulus lupulus* Linne., also called hop plants. Combination of xanthohumol (50 μM) and TRAIL induced the cleavage of caspase-8, -9, and -3, activated Bax in LNCaP cells. These findings demonstrated the capacity of xanthohumol as an antitumor agent when used with TRAIL [53]. 3-butenyl isothiocyanate (BITC) is a dietary compound isolated from *Brassica juncea* Linne. Czen. Increased level of c-caspase-3 and decreased MMP were shown in BITC treated PC3 prostate cancer cells [54]. 6α-acetoxyanopterine was extracted from *Anopterus macleayanus* F. Muell., an endemic Australian tree. This dietary compound (1.25, 2.5, and 5 nM) induced the cleavage of PARP only against LNCaP cells [55].

Some of the remaining dietary substances were discovered from fungi (Table 2). Artepillin C is a dietary compound derived from cinnamic acid extractions from propolis, a resinous substance produced by honeybees. The formation of DNA fragmentation was shown in artepillin C-treated prostate cancer cells. Meanwhile artepillin C (50 μM) increased the level of c-PARP, c-caspase 3 [56]. Diketopiperazine disulfide glionitrin A is a natural product extracted from *Aspergillus fumigatus* Fresen. The treatment of this compound (0.75, 1.5, and 3 μM) upon DU145 cells induced the activation of caspase-3, -8, and -9, c-PARP, and Bax, while Bid and PARP expression were decreased. It was further reported that in five-week-old male BALB/c-nu mice treated with glionitrin A (5, 10 mg/kg) were shown with decreased tumor size compared to the control group [57]. D-Trp isomerized CJ-15, 208 (cyclo (Phe-D-Pro-Phe-Trp)) is a dietary compound isolated from the fermentation broth of a fungus. Treatment of this compound (10 μM) induced apoptosis in PC3 cells [58]. Malformin A1 is a dietary substance extracted from *Aspergillus niger* Tiegh. The compound (150 nM) triggered the activations of c-caspase-3, and c-PARP, meanwhile a decrease of the level of Bcl-2 in PC3, and LNCaP cells [59]. Viriditoxin is a dietary compound isolated from *Paecilomyces variotii* fungus, which was derived from the jellyfish *Nemopilema nomurai*. This compound (0.1, 0.5, and 1 μM) activated the c-PARP, Bax, cytochrome c, and c-caspase-3, and decreased the level of Bcl-2 in LNCaP cells [60].

**Figure 1:**
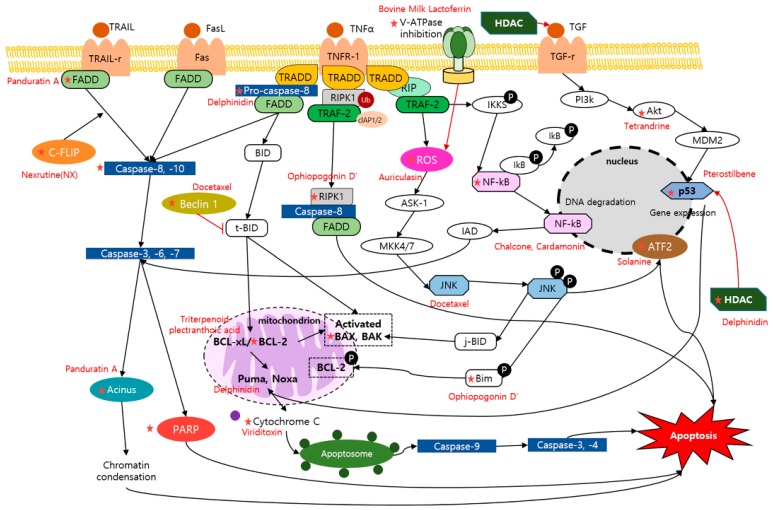
Schematic diagram of apoptotic mechanisms of dietary compounds. ★, The molecule is regulated by the compounds.

There are other dietary compounds originated from un-known sources or unique living organisms or organic matter (Table 3). Heteronemin is a marine sesterterpenoid-type dietary product, which is derived from *Hyrtios* sp., and is known to possess various bioactivities including the apoptotic effect [61]. Heteronemin (2.56, 5.12 μM) induced apoptosis through up-regulation of c-caspase-3, c-PARP in LNCaP, and PC3 cells. Furthermore, a significant decrease in tumor size of LNCaP tumor xenograft animal model was reported [62]. Bovine milk lactoferrin, a dietary protein extracted from milk, demonstrated a potential therapeutic activity against highly metastatic cancer cell line, PC3. The BLf (175 μM) induced the exposure of phosphatidylserine in PC3 cells. Moreover, the percentage of early and late apoptotic cells of PC3 were increased by BLf exposure [63]. δtocotrienol and γ tocopherol are substances derived from the Vitamin E family. The two substances (10 μM) showed apoptotic activities against LNCaP cells, inhibiting the cell growth, and inducing synergistic effects in apoptosis [64]. Dioscin is a dietary product that possesses various clinical effects. The treatment of dioscin on PC3 cells (5.6, 10 μM) resulted in an up-regulation of c-PARP, c-caspase-3, and Bax, and down-regulation of Bcl-2. Additionally, it was elucidated to have suppressive effect on the tumor growth in a PC3 xenografts model in a dose of 80 mg/kg [65]. LLDT-288 (1, 10 μM) is known to have cytotoxic activity upon human prostate cell lines. It was demonstrated that activated c-PARP, c-caspase 3, and Bax but inhibited NF-κB, caspase-3, -9, and Bcl-2 were shown in LLDT-288-treated PC3 cells. Furthermore, the results of PC-3 xenograft mice models showed that LLDT-288 (5, 10, and 20 mg/kg) induced the apoptotic effect [66]. Pterostilbene is a dietary analog of resveratrol, having metastasis-associated protein 1 (MTA1)-targeted chemopreventive and therapeutic efficacy upon prostate cancer cells. When treated to prostate-specific heterozygous (Pten+/f) and Pten-null (Ptenf/f) mice (10 mg/kg), it increased the activity of c-caspase3, p21 and p27 in LNCaP, and DU145 cells, and p53 acetylation with an increase in Bak. These findings showed the possibility of combinational strategies with Pterostilbene in treatment of prostate cancer cells [67]. The mechanism of dietary compounds reviewed were illustrated and arranged in Figure 1 and Table 4.

### 2.2. Anti-Angiogenesis and Dietary Compounds

**Figure 2:**
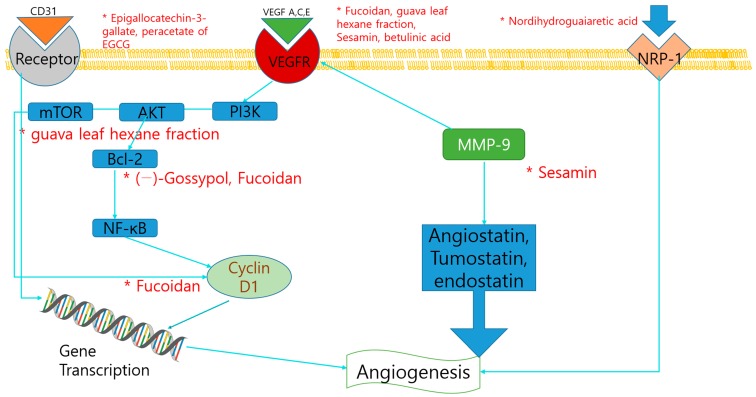
Schematic diagram of anti-angiogenic mechanisms of dietary compounds. *, The molecule is regulated by the compounds.

Angiogenesis, or neovascularization, is essential to tumor survival and proliferation. Anti-angiogenic therapy has become an important area of research within the last decade in the treatment of cancer. Several studies indicated the anti-angiogenesis effect and mechanism of dietary compounds (Table 5). Fucoidan, is a sulfated polysaccharide obtained mainly in various species of brown algae and brown seaweed, is reported to have anti-tumor activity against lung, breast, liver, colon, prostate, and bladder cancer cells. It shows antiangiogenic effects by downregulation of the JAK-signal transducer and activator of transcription 3 (STAT3) pathway and STAT3-regulated genes, such as VEGF, Bcl-xL, and cyclin D1 [68]. Nordihydroguaiaretic acid, a phenolic compound extracted from creosote bush (*Larrea tridentate*), inhibited the NRP1 expression in PC3 cells, which is a co-receptor of VEGF [69]. Terrestrosin D is a major steroidal saponin derived from *Tribulus terrestris* L., which is a medicinal plant distributed widely in the Mediterranean. It induced the cell growth arrest and apoptosis by inhibition of NF-κB signaling in liver cancer cells, and that it exhibited the weak cytotoxic effects to normal cells compared to cancer cells. Terrestrosin D inhibited the angiogenesis in PC3 and HUVEC cells, by direct downregulation of endothelial cells proliferation although it increased the VEGF secretion as observed in ELISA analysis [49]. Resveratrol and pterostilbene, a dimethylether analogue of resveratrol, are reported to have anti-angiogenic properties by regulating MTA1, involved in processes related to metastasis, such as invasion and angiogenesis. Importantly, pterostilbene was more potent than resveratrol in mediating an increase in p53 acetylation through inhibition of MTA1. MTA1-knockdown cells were also sensitized by resveratrol and pterostilbene treatment [70]. Guava (*Psidium guajava* Linn.) leaves are rich in triterpenoids, flavonoids, essential oil, and tannins and are known to exhibit anti-inflammatory, spermatoprotective, and chemopreventive effects. Since overexpression of AKT/mTOR/S6K1 signaling pathway is closely linked with angiogenesis in prostate cancer, the effects of guava leaf hexane fraction on this pathway was observed. As a result, guava leaf not only induced significant apoptotic effects, but also suppressed constitutive activation of PI3K/AKT/mTOR/S6K1 in PC3 cancer cells. Consistent with this evidence, the research also showed that guava leaf could clearly suppress the VEGF expression at a lower concentration of 50 μg/mL [71]. Epigallocatechin-3-gallate (EGCG) is one of the major polyphenol compounds of green tea that has been reported to have anticancer effects against various types of cancers, including prostate cancer. EGCG and its synthetic derivative, the peracetate of EGCG (EGCG-P), were used to investigate the inhibitory effects on CWR22R prostate tumor xenografts. The antiangiogenic effect of the compounds was observed in immunohistochemistry (IHC) staining of CD31, and as a result, alteration in microvessel density was exhibited [72]. Betulinic acid is a pentacyclic triterpene dietary product initially known to have antiangiogenic responses in tumors without identifying the underlying mechanism. It is demonstrated that betulinic acid decreased the expression of VEGF and the antiapoptotic protein survivin in LNCaP cells. Activation of selective proteasome-dependent degradation of the transcription factors specificity protein 1 (Sp1), Sp3, and Sp4 regulated VEGF and surviving activation [73]. (–)-Gossypol, a natural polyphenol from cottonseed, has been identified as a potent inhibitor of Bcl-2 and Bcl-xL. Anti-CD31 immunohistochemical staining showed that (–)-gossypol plus radiation significantly inhibited tumor angiogenesis, thus indicating that it could radiosensitize prostate cancer in vitro and in vivo without exacerbating toxicity [74]. The anti-angiogenesis mechanisms of dietary compounds were illustrated in Figure 2. 

### 2.3. Anti-Metastasis and Dietary Compounds

**Figure 3:**
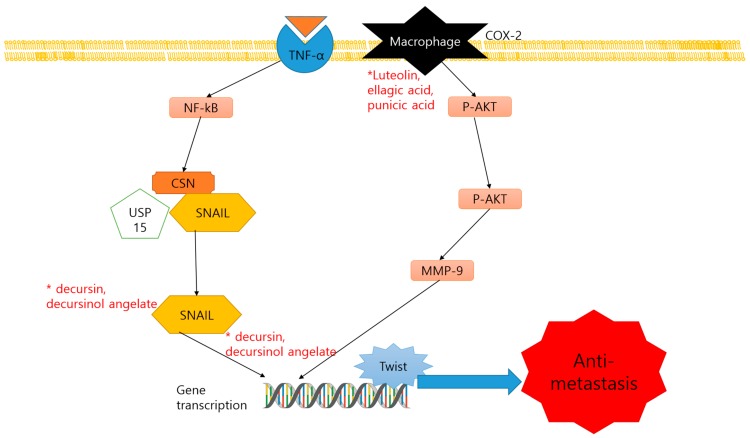
Schematic diagram of anti-metastatic mechanisms of dietary compounds. *, The molecule is regulated by the compounds.

Metastasis is responsible for a majority of cancer-related deaths. Tumor invasion to the surrounding tissue and metastasis are resulted from the multistep processes that include proteolytic degradation of the surrounding extracellular matrix (ECM), allowing malignant cells to move into and through the ECM. The epithelial-to-mesenchymal transition (EMT) is the crucial step for cancer cells to initiate the metastasis. Two studies of dietary compounds were reported their anti-metastatic effect in prostate cancer cells (Table 6). Decursin and its structural isomer, decursinol angelate are the major pyranocoumarin compounds of the alcoholic extract of the *Angelica gigas* Nakai root. They have been reported as an effective anticancer strategy by inhibiting the growth of prostate epithelium and neuroendocrine carcinomas in the transgenic adenocarcinoma of mouse prostate (TRAMP) model. Profiling of N-ethylcarboxamideadenosine (NE-Ca) mRNA showed an anti-invasion-metastasis effect by inhibiting Snail2, Twist, Notch1, and Tgfbr2, in the prostate cell line and repressing the Snail2 in Tramp mice [76]. Luteolin (L), ellagic acid (E), and punicic acid (P) are major compounds found in pomegranates. They slowed the tumor progression by inhibition of the PLA2, COX, and AA metabolism pathway. The antimetastatic effect of the compounds is proved by examining the protein levels of CXCR4. As a result, these components exhibited the down-regulation of CXCL12/CXCR4 axis in vivo, and its signaling pathways Gα13, phosphoinositide 3-kinases (PI3K) and p-AKT [77]. The anti-metastatic mechanisms of dietary products were illustrated in Figure 3.

### 2.4. MiRNA Regulation and Dietary Compounds

MiRNAs are involved in the cancer progression by regulating the cancer related-molecular events including apoptosis, angiogenesis, and cell cycle control. Modulation of mi-RNAs through dietary products can exert chemoprotective effects on various types of cancers [78] (Table 7). Major compounds of pomegranate juice, luteolin, ellagic acid, and punicic acid showed enhanced the effects on metastasis through miRNA modulation. In miRNA PCR array analysis, luteolin, ellagic acid, and punicic acid treatment elevated the various anti-tumor miRNAs, including miR144, miR-133b, miR-1, miR-122, etc. [79]. EGCG is polyphenol in green tea, which functions as a direct antagonist of androgen action, modulating the expression levels of miRNAs in tissues from PC3 xenografts model. A notable down-regulation of androgen-regulated miRNA-21 and up-regulation of a tumor suppressor, miRNA-330, in cancers of EGCG treated mice were observed [80]. Green tea polysaccharide is derived from *Camellia sinensis* L. and was elucidated to have anti-tumor effects against prostate cancer by down-regulation of mi-R93, which was overexpressed in prostate cancer patients. These dietary compounds also induced the apoptosis by the increase of Bax/Bcl-2 ratio and c-caspase-3 protein expression [81]. Swati et al. reported that resveratrol, a well-known anti-cancer natural compound, regulated fifty one cancer related miRNAs according to the miRNA microarrays. Among them, phosphatase and tensin homolog deleted onchromosome 10 (PTEN)-targeting miRNAs, including miR-17, miR-20a, miR-20b, miR-106a, and miR-106b (miR-17-92 and miR-106ab cluster) were down-regulated. PTEN expression was also up-regulated by the resveratrol treatment and carcinogenesis of prostate cancer was repressed [82]. Genistein is a dietary compound included in numerous plant sources such as lupin, fava beans, and coffee. MiR-1260b was more frequently observed in prostate cancer tissues compared to benign prostate hyperplasia. The miR-1260b was significantly decreased with the administration of genistein. Two tumor suppressor genes, sFRP1 and SMAD, were up-regulated by genistein as a consequence of direct regulation of miR-1260b [83]. Curcumin is a polyphenol from *Curcuma longa,* which is reported to prevent the development of prostate cancer in TRAMP mice as a DNA hypomethylation agent [84]. EF24, one of the many kinds of curcumin analog, showed the enhanced anti-carcinogenic reactions related to NF-κB and miRNA-21 [85]. Through DNA methylation, curcumin activated the silenced Nrf2 gene epigenetically. Soy phytoestrogens, genistein, and daidzein showed the protective effect of soy against prostate cancer by promoting demethylation of GSTP1 and EPHB2 promoter regions in prostate cancer cells [86]. Simultaneous intake of gefitinib and luteolin also induced the increased the expression of miR-630 and miR-5703 in PC-3 cells, exerting cell cycle arrest of prostate cancer [87].

### 2.5. Multi-Drug Resistance (MDR) and Dietary Compounds

Multi-drug-resistance (MDR) is a major cause of failure in cancer chemotherapies, which also significantly lowers the recovery rate of cancer patients. Various mechanisms related to the decrease of sensitivity of anti-cancer drugs including ATP-binding cassette transporter family, apoptosis induction, autophagy induction, cancer stem cells, miRNAs hypoxia induction, and epigenetic regulation [78]. Number of compounds derived from dietary products have been suggested as potential solutions to drug resistance (Table 8). 6-gingerol, 10-gingerol, 6-shogaol, and 10-shogaol are derived from ginger, demonstrating the anti-tumor effects by suppression of multidrug resistance-associated protein 1 (MRP1) and glutathione S-transferase Pi (GSTπ) protein expression [33]. Scutellarin, which is glycoside isolated from *Scutellaria altissima* L., which has been used to treat pneumonia, upper respiratory infection, and high blood pressure. The research demonstrated that this compound significantly sensitized PC3 cells to cisplatin, which is one of the major chemotherapeutic agents by exerting DNA damages. The higher level of γ-H2AX expression in the immunofluorescence focus assay was exhibited in GSTπ-exposed PC3 cells [47]. (8R)-3β,8-dihydroxypolypoda-13E,17E,21-triene is a polypodane-type bicyclic triterpenoid isolated from *Pistacia lentiscus* oleogum resin, stimulating the apoptosis in androgen-independent and chemoresistant PC3 prostate cancer cells both in vitro and in vivo. This compound increased the accumulation of the cells in G0/G1 stage while decreased accumulation in the S phase, whereas docetaxel induced the cell accumulation in G2/M phase [88]. Theaflavins, epicatechin-3-gallate, and epigallocatechin-3-gallate derived from black and green tea sensitized the anti-cancer effect of docetaxel by downregulation of MDR-1 expression [89].

## 3. Discussion 

Prostate cancer is one of the most common tumor types in middle and older aged men [1]. Although several studies have been conducted to find optimal treatment methods, conventional chemotherapeutic agents showed various side effects such as urinary incontinence, erectile dysfunction, libido loss, bowel problems, breast changes, hot flushes, and fatigue [90]. Thus, the novel methods of therapies are needed, and natural materials have received great interest in this field. In this study, numerous dietary products with antitumor activities against prostate cancer were reviewed. To clarify the causative agent of the anticancer effect, the subject of our research were limited to single dietary compounds. We classified the mechanisms of each dietary compounds into five categories; apoptosis, anti-angiogenesis, anti-metastasis, miRNA regulation, and anti-MDR, which were based on the types of mechanism that suppressed the pathogenesis of prostate cancer. Reflecting the latest research on the mechanism of cancer, we investigated single dietary compounds that exerted anti-prostate cancer activities.

Apoptosis is an important mechanism in regulating homeostasis of cell survival. The defect of this activity is a critical factor of tumor pathogenesis. Thirty-nine dietary compounds were collected and proved to have anti-apoptotic effects against prostate cancer. Studies were mostly dealt with LNCaP, DU145, or PC3 prostate cancer cell lines, with some research including in vivo studies. Induction of apoptosis of the dietary compounds was measured by several factors such as Bcl-2, Bax, PARP, caspases, and c-FLIP. Especially, caspase and PARP cleavage were two main factors that caused apoptosis in prostate cancer cells, which were related with thirty-three and twenty one dietary compounds each. Furthermore, several reports elucidated the apoptotic effects of dietary substances in both cell line and in vivo studies [39,42,48,49,57,62,65,66]. These studies analyzed the chemical mechanisms as well as verifying actual reactions in organism. Although most reports clearly demonstrated the correlation of dietary compounds and their antitumor effects, some studies only displayed apoptosis phenomena, without specific mechanisms [29,34,46,58,64]. Additionally, a couple of studies administrated abnormally high doses of extracts upon cancer cell lines, both treated with more than 100 μM of compounds [30,47]. They may be toxic not only in cancer cells but also in human normal cells at high concentration, additional experiments for measuring stability.

Angiogenesis, a hallmark of antitumor activity, acts abnormally in cancer formation. Nine compounds were collected to have anti-angiogenesis-related mechanisms. Six of the dietary compounds down-regulated the VEGF expression, an important activation factor of angiogenesis. A study with betulinic acid was conducted with both LNCaP prostate cancer cells and LNCaP xenograft models, and it specifically addressed its anti-angiogenetic effects by experimenting with various concentrations [73]. On the other hand, several studies only demonstrated a single mechanism, which was not even an essential factor in the occurrence of angiogenesis, such as the inhibition of Bcl-2 by (−)-Gossypol treatment [74]. These compounds also showed the lower efficacy in the inhibition of angiogenesis in prostate cancer cells.

Metastasis is also an important mechanism in the progress of cancer and cancer-related death. We have collected three compounds that resisted metastasis in prostate cancer cells. Decursin, decursinol angelate, and anthocyanin displayed inhibitory activities of Snail 2 [76]. Snail is an inducer of EMT, which is positively involved in a metastatic cascade of many solid tumors [91]. These two studies adapted various cell lines and confirmed diverse mechanisms that prevented metastasis. Especially, decursin and decursinol angelate also showed the several other mechanisms such as the down-regulation of twist, notch, and E-cadherin. The TWIST family regulates the EMT transcriptome program and are also known to induce the apoptosis. The notch family, which is signaling pathway that mediate the stem cell renewal and regulate EMT. Besides, E-cadherin is one of the most prominent epithelial proteins that characterized EMT and intervenes with activities in the growth and metastasis of cancer [92]. These various mechanisms demonstrated the potentiality of the anticancer effects of the proposed dietary compounds.

MiRNAs are important biomarkers for diagnosis, prognosis, and therapeutic purposes in cancer [93]. Recent studies indicated that miRNA expression and circulation in significantly associated with metastasis and clinicopathological end points in prostate cancer patients [94]. Let-7 families, miR-99a, miR-141, miR-198, and miR-205 are marked as related miRNAs that showed different expressions in prostate cancer cells [95]. Our research found that luteolin, ellagic acid, punicic acid epigallocatechin-3-gallate, and green tea polysaccharides exhibited antitumor effects through miRNAs regulation [79,80]. Especially, luteolin, ellagic acid, and punicic acid regulated the expression of let-7 families and miR-15a, which are known as tumor suppressive miRNAs [96]. Although the discussion of miRNA and its correlation to cancer has been progressed, there are limitations in utilizing miRNAs in clinical treatment regarding bio instability and off-target effects [93]. At the same time, research on the ways in which dietary compounds could regulate miRNAs is a field that required further investigation.

Lastly, multi-drug resistance is one of the crucial factors that disrupt the efficacy of medicinal treatment of cancer [97]. Previous studies have suggested various chemotherapeutic agents that could reverse MDR, but toxic responses and the decrease in clear efficacy due to an interaction with other pharmaceuticals were the problems. As a result, natural compounds such as tetrandrine and quercetin were referred as a source for the next generation that could solve current problems with multidrug resistance [98,99]. In this study, four studies were reviewed about multidrug resistance agents for prostate cancer. 6-gingerol, 10-gingerol, 6-shogaol, and 10-shogaol were reported to decrease the expression of MRP1, a protein transporter that controls drug efflux from cancer cells [33,100]. Moreover, two compounds showed the expression of MDR genes, including MDR-1 and GST, which are correlated with drug tolerant tumor or metastasis [101,102]. New discoveries of dietary compounds targeting MDR of prostate cancer can be effective measures for the treatment of prostate cancer. Meanwhile, a study conducted with scutellarin, an excessively high dose (200 µM) was used, and its adverse toxic effects were yet to be discovered [47]. Due to the lack of clinical evaluation system and biomarkers, evaluation of multidrug resistance of dietary compounds are limited to in vitro studies. Furthermore, drug interaction between chemotherapeutics and dietary compounds are also factors that require further studies to be continued. However, the role of phytochemicals as epigenetic modulators in regulating carcinogenesis is gaining evidence. Green tea polyphenols were discovered to reactivate tissue inhibitors of MMPs, which are known to accelerate the proliferation of prostate cancer [103]. These findings showed the potential of dietary compounds as effective epigenetic inducers. Yet, additional research needs progress in order to resolve the limitations of establishing specific pharmacokinetic and pharmacodynamics profiles in human administration [104].

Among the discoveries, two dietary compounds showed two different efficacies against prostate cancer. Scutellarin had both apoptotic and multidrug resistance [47]. Terrestrosin D induced the apoptosis and inhibited the angiogenesis through decrease of endothelial cells proliferation [49]. These discoveries suggest the potential of dietary compounds as an active agent against prostate cancer.

Dietary compounds are potential anti-cancer agents that have protective and therapeutic effects with less side effects. Vegetable and fruits are found to reduce advanced or aggressive prostate cancer, and phytochemicals like lycopene are commonly known to suppress prostate carcinogenesis [105]. Tomato and broccoli are effective dietary ingredients that show anti-tumor activities, including the decrease of tumor proliferation and an increase of apoptosis. Additional effects such as the regulation of serum testosterone levels and tumor size represent the potential effects of dietary therapy for treating prostate cancer [106]. Furthermore, dietary compounds interact with multiple targets, hence can have synergistic anti-cancer effects with conventional therapeutic agents [107]. Based on these reports about the potential effects of natural products, we conducted a new review on dietary compounds that are effects in prostate cancer. Consequently, over seventy dietary compounds were confirmed to obtain antitumor effects upon prostate cancer. Previous studies were only focused on the effects of one type of dietary compound, such as thymoquinone and resveratrol [108,109]. We categorized the compounds according to their anti-tumor mechanisms in order to elucidate their specific pharmaceutical pathway. In other cases, research has shown how one substance can affect various types of cancer, or how it affects a variety of mechanisms [110]. By focusing on one cancer type, our present review attempted to provide a sufficient database of dietary compounds that could be selected as options for alternative treatments of prostate cancer. Furthermore, the different mechanisms that are related to each compound could provide pharmacological evidence in the use of these compounds as anti-cancer agents.

One of the limitations of this study is that it excluded crude extracts and mixtures of various plant materials. In the medical field, various dietary substances have been used as anticancer drugs, and their forms were variable from a single compound to a complex extract. They were often used in combination with existing chemotherapy. Randomized phase II trial of docetaxel and thalidomide against prostate cancer was conducted with seventy-five patients from 1999 to 2001, demonstrating that docetaxel could increase its therapeutic efficacy through combination with thalidomide [111]. There also has been a randomized phase II study regarding the immunological efficacy of herbal medicines including Hochu-ekki-to and Keishi-bukuryo-gan in combination with personalized peptide vaccination (PPV) for castration-resistant prostate cancer from 2008 to 2014 [112]. The results showed that monocytic myeloid-derived suppressor cells (Mo-MDSC) frequency and IL-6 level were more stabilized by the medicines. Besides, several herbal medicines have shown their effectiveness in reducing side effects of immunotherapy in cancer patients. However, clinical studies still face various limitations such as funding problems and the lack of intervention studies from laboratory studies to clinical research [113]. Considering this clinical background, more detailed studies on anti-prostate cancer effects of dietary substances are necessary.

Even though researches on dietary compounds have progressed enough to make a significant number of outcomes, they still face a variety of difficulties in standardization, drug stability, and toxicity. The difficulty of isolating of active constituents and confirming their targeted effects is one of the problems in standardizing dietary compounds. Results from studies with the same substance may also vary due to the difference in the quality and quantity of materials that are collected. Moreover, the reported effects of natural compounds relatively lack its reproducibility. Depending on the types and conditions of experiments, drugs can result in different consequences. For instance, apigenin is a plant flavone that has been demonstrated to have multiple anti-tumor effects on various cancer types including prostate cancer. However, due to its reduced solubility in water, when administrated in clinical approaches, oral bioavailability was lower compared to previous in vivo and in vitro studies [114]. Another problem of the development of new pharmaceuticals is the unexpected side effects. Docetaxel is a natural compound that showed pro-apoptotic effects related to PARP, beclin 1, and JNK regulations [32]. However, there are also reports about side effects such as neutropenia, hypersensitivity problems, and fluid retention [115]. Although these problems were mentioned to be relieved by supportive measures, further research is necessary to resolve these problems. Hence, safety and stability of dietary compounds are other important matters to consider in case of the possibility of adverse toxic effects. Although dietary compounds in the food have less side effects, but consumption of high concentration of one compound could induce adverse toxic effects. Thus, combination therapy with higher efficacy without side effects could be a solution for the development of novel drugs for cancer. This review could be a reference for those who want to find potent materials to combine for cancer treatment.

The ultimate goal of research on dietary substances is to find effective supplements that prevent and reduce the risks of prostate cancer and reduce the side effects of conventional cancer treatment. In order to utilize these compounds in actual clinical use, additional studies are necessary to select dietary compounds with appropriate doses that have the greatest effect and minimal toxicity when administered to humans. Experiments are also needed to determine reactions that may occur in combination with conventional chemotherapy or with other various natural substances. We expect further research to select representative substances that show outstanding anticancer effects, and to verify the effects on various conditions. Taken together, this study could be base research for data on the discovery of new prostate cancer antitumor drugs.

## 4. Methods

Researches regarding the effects of dietary products on prostate cancers were collected from PubMed. Upon searching for relevant studies, prostate cancer, dietary compound, natural product, apoptosis, metastasis, MDR, and miRNA were used as keywords. Then we collected studies that fit into the criteria: (1) Researches based on in vitro or in vivo to prove anticancer effects of dietary product (2); researches that demonstrated reliable statistical analysis data (*p* values that were less than 0.05); (3) researches that were not upset by subsequent studies; and (4) researches that include experiments with natural-derived compounds. The family names of dietary products and herbs mentioned in this review are based on a reliable source. Dietary product-derived compounds in this review were double-checked from the NCBI PubChem website for precision.

## 5. Conclusions

Our study reviewed the single dietary compounds that showed anti-tumor activities against prostate cancer. The dietary compounds showed anti-cancer efficacies through several mechanisms and the possibility of natural products is demonstrated as important sources of advance control and treatment for prostate cancer. We expect that the results will be utilized as an academic foundation for developing novel drugs with less side effects that can be practically used in dietary supplement as well as clinical treatments.

## Figures and Tables

**Table 1:** Apoptosis inducing dietary compounds originated from plants.

Compound	Classification	Source	Organism	Cell Line/Animal Model	Dose; Duration	Mechanism	Reference
Auriculasin	Isoflavonoids	*Flemingia philippinensis* Merr. and Rolfe	Plant	LNCaP	5 μM/24 h	Bax, c-PARP, ROS↑	[27]
						Bcl-2↓	
Chalcone Cardamonin	Flavonoids	*Campomanesia adamantium* Myrtaceae	Plant	PC3	11.35 μg/mL/12, 24, 48 h	NF-kB ↓	[28]
Cyclohexenyl chalcone panduratin A (PA) and nicolaioidesin C (NC)	Flavones	*Boesenbergia pandurate* Roxb	Plant	PC3, DU145	5, 10, 20 μM/2 days		[29]
Delphinidin	Anthocianidin	Pomegranates, berries, grapes, beets, and eggplants	Plant	(1) LNCaP	50, 100, 150 μM/24 h	(1) c-caspase-3, -7, c- PARP, c-HDAC3, Puma, Bax, Noxa ↑	[30]
				(2) DU145		Pro-caspase-8, HDAC3 ↓	
				(3) PC3		(3) HDAC3↓	
Dimeric ellagitannins (cornusiin A, camptothin B, C_75_H_56_O_48_ (amorphous))	Tannins	*Cornus alba* Linne.	Plant	(1) DU145	(1) 20, 50, 100 μM/48 h		[31]
				(2) LNCaP	(2) 5, 10, 20 μM/48 h		
Docetaxel	Flavones	*Taxus baccata* L.	Plant	PC3, DU145	100 ng/mL/12 h, 24 h	c-PARP, p-Bcl-2, Beclin1, p-JNK↑	[33]
	(semisynthetic)						
Emodin, rhapontigrnin	Anthraquinones	*Rheum palmatum* Linne.	Plant	DU145	50 μM/48 h		[34]
FD1a, FD1c, FD1h, FD2a, FD2c, FD2h	Flavonoids	*Ficus deltoidea* Linne.	Plant	(1) PC3	(1) 19 mg/mL/72, 96 h	(1) c-caspase 3, 7, Bax, Bcl-2 ↑	[35]
				(2) LNCaP	(2) 23 mg/mL/72 h	(2) c-caspase-3, -7 ↑	
Flavonoid	Flavonoids	*Diospyros kaki* Linne.	Plant	PC3	12.5, 25 μg/mL/24 h	Bax, c-caspase-3↑	[36]
						Bcl-2↓	
Guttiferone F	Benzophenones	*Allanblackia stuhlmannii* Engl.	Plant	(1) PC3	10 μM/6, 12, 24 h	(1) & (2) c-caspase-9, −7, −3, c-PARP ↑	[37]
				(2) LNCaP		caspase-9, -7, -3, t-PARP ↓	
						(2) Bcl-2, Bcl-2/Bax ↓	
Jungermannenone A and B	ent-kaurane diterpenoids	*Jungermannia fauriana* Beauverd.	Plant	PC3	JA: 1.5 μM/L/12, 24, 48 h	c-caspase-3, c-PARP ↑	[38]
					JB: 5 μM/L/12, 24, 48 h		
Linalool, Linalyl acetate	Flavonoids	*Lavandula angustifolia* Mill.	Plant	(1) PC3, DU145	(1) 2.5 µM/24 h		[39]
				(2) Nude mice PC-3 prostate cancer cell xenograft model	(2) 10, 25, 50, 100, 200 mg/kg/7 days		
Matrine, Oxymatrine, Sophocarpine, Xanthohumol	Alkaloids	*Sophorae flavescens* Aiton	Plant	PC3	0.1–2 mg/mL/72 h		[40]
Nexrutine (NX)	Alkaloids, Phenolic compounds, flavone glycosides	*Phellodendron amurense* Rupr.	Plant	PC3	10 μM/72 h	c-caspase-3 ↑	[41]
						c-FLIP ↓	
Ophiopogonin D	Steroidal Glycoside	*Ophiopogon japonicus* Linne. f.	Plant	(1) PC3, DU145	(1) 2.5, 5.0 μM/6h	(1) RIPK1, Bim ↑	[42]
					(2) 2.5 or 5.0 mg/kg; 24 days (5 days a week)	c-RIPK1, c-caspase-8, -10, Bid↓	
				(2) BALB/c nude xenograft mice			
Panduratin A	Flavones	*Kaempferia pandurata* Roxb.	Plant	(1) PC3, DU145	(1) 20 μM/24 h	(1) Bax, FADD, TRAIL, c-PARP ↑	[43]
						Bcl-2, Bid, acinus, t-PARP, pro-caspase -3, -6, -8, -9 ↓	
Punicalagin	Ellagitanin	*Punica granatum* Linne.	Plant	(1) PC3	(1), (2) 100 μM/24 h, 12 h	(1) c-caspase-3, -8 ↑	[44]
				(2) LNCaP			
Physalin A, B	Steroids	*Physalis alkekengi* Linne.	Plant	C42B, CWR22Rv1	10 μM/12, 24, 48 h, or 5, 10, 16 μM/24 h	t-PARP, pro-caspase 3 ↓	[45]
Schizandrin (1) derivatives compound 5	N/A	*Schisandra grandiflora* Wall.	Plant	DU145	1, 2 μM/48 h		[46]
Scutellarin	Flavone	*Scutellaria altissima* Linne.	Plant	PC3	200, 400, 600 μM/24 h	c-caspase-3, -9, Bax↑	[47]
						MMP, Bcl-2, ↓	
Solanine	Glycoalkaloids	*Solanum nigrum* Linne.	Plant	(1) DU145	(1) 10–160 μM/L/1 h	(1) P38 pathway, p-ATF2, Bax↑	[48]
				(2) Six-to eight-week- old male nude mice with subcutaneous DU145 prostate cancer cell xenografts	(2) 5 mg/kg/4 weeks	Bcl-2 ↓	
Terrestrosin D	Steroids	*Tribulus terrestris* Linne.	Plant	(1) PC3	(1) 2, 5 μM/24 h	(1) MMP ↑	[49]
				(2) Male nude mice (5 weeks of age, BALB/c)	(2) 25, 50 mg/kg/4wks (3 times a week)		
Tetrandrine	Alkaloids	*Stephania tetrandra* S. Moore	Plant	DU145, PC3	2.5, 5.0 10.0 μM/48 h	c-caspase-3, c-PARP, Bax↑	[50]
						Akt, Bcl-2 ↓	
Thalicthuberine	Alkaloids	*Hernandia albiflora* Kubitzki	Plant	(1) LNCaP,	(1) 2.5 μM/48h	(1) c-caspase-3, -7 ↑	[51]
				(2) CEM	(2) 5, 10 μM/24 h	(2) caspase-3, -7 ↑	
				(3) VCR-R	(3) 60 μM/24h	(3) caspase-3, -7 ↑	
Triterpenoid plectranthoic acid (PA)	Hopanoids	*Ficus microcarpa* Linne.f.	Plant	DU145, PC3, NB26	20, 40 μM/24 h	Bax, c-PARP, c-caspase3 vinculin ↑	[52]
						Bcl-xl, Bcl-2 ↓	
Xanthohumol	Prenylated Chalconoid	*Humulus lupulus* Linne.	Plant	LNCaP	50 μM/2, 8 h	c-caspase-8, -9, -3, Bax↑	[53]
						(Combination of TRAIL)	
3-butenyl isothiocyanate	Isothiocyanate	*Brassica juncea* Linne. Czen	Plant	PC3	0.041 μL/mL, 0.060 μL/mL/12-14 h	c-caspase-3 ↑	[54]
						MMP ↓	
6α-acetoxyanopterine	Alkaloids	*Anopterus macleayanus* F.Muell.	Plant	(1) LNCaP	1.25, 2.5, 5 nM/24 h	(1) c-PARP↑	[55]
				(2) PC3			

N/A, not available; ↑, up-regulated; ↓, down-regulated.

**Table 2:** Apoptosis inducing dietary compounds originated from fungi.

Compound	Classification	Source	Organism	Cell Line/Animal Model	Dose; Duration	Mechanism	Reference
Artepillin C	Phenolic acid	Propolis	Fungus	CRPC CWR22Rv1	50 μM/24 h	c-PARP, c-caspase-3 ↑	[56]
Diketopiperazine disulfide glionitrin A	Diketopiperazine metabolite	*Aspergillus fumigatus* Fresen. Sphingomonas sp. (KMK-001)	Fungus	(1) DU145	(1) 0.75, 1.5, 3 μM/24 h	(1) c-caspase-8, -9, -3, c-PARP, Bax ↑	[57]
				(2) Five weeks-old male BALB/c-nu mice (17–22 g) b earing xenografts of DU145 c ells	(2) 5, 10 mg/kg/27 d	Bid, PARP ↓	
D-Trp isomerized CJ-15, 208	Macrocyclic Peptide	Fungus	Fungus	PC3	10 μM/24, 48 h		[58]
(cyclo [Phe-D-Pro-Phe-Trp])							
Malformin A1	Quassinoids	*Aspergillus niger* Tiegh.	Fungus	PC3, LNCaP	150 nM/6, 12, 24 h	c-caspase-3, c-PARP ↑	[59]
						Bcl-2 ↓	
Viriditoxin	N/A	Paecilomyces variotii (*Bissochlamys spectabilis* Udagawa and Shoji Suzuki)	Fungus	LNCaP	0.1, 0.5, 1 μM/48 h	c-PARP, Bax and cytochrome c, c-caspase-3 ↑	[60]
						Bcl-2 ↓	

N/A, not available; ↑, up-regulated; ↓, down-regulated.

**Table 3:** Apoptosis inducing dietary compounds originated from other organisms.

Compound	Classification	Source	Organism	Cell Line/Animal Model	Dose; Duration	Mechanism	Reference
Heteronemin	Sesterpenoid	*Hyrtios* sp.	Metazoa	(1) LNCaP, PC3 (2) Male immunodeficient athymic mice	(1) 2.56, 5.12 μM/24 h (2) 1 mg/kg/29 days	GAPDH, c-caspase -3, c-PARP↑	[62]
Bovine Milk Lactoferrin	Glycoprotein	Milk	Animal	PC3	175 μM/48 h or 72 h	V-ATPase↑	[63]
δTocotrienol, γ tocopherol (each and combined)	Tocotrienol (vitamin E)	Vitamin E	N/A	LNCaP	10 μM/48 h		[64]
Dioscin	Spirostanyl glycoside, Hexacyclic triterpenoid	N/A	N/A	(1) PC3 (2) PC3 cell tumor xenograft model	(1) 5.6, 10 μM/24 h (2) 80 mg/kg/28 days	(1) c-PARP, c-caspase-3, Bax ↑ Bcl-2 ↓	[65]
LLDT-288	Triptolide analogue	N/A	N/A	(1) PC3 (2) Human prostate (PC-3) xenograft mice model	(1) 1, 10 μM/36 h (2) 5, 10, 20mg/kg, PO, BID/21 days	(1) c-PARP, c-caspase-3, Bax ↑ NF-kB, caspase-3, -9, Bcl-2 ↓	[66]
Pterostilbene	Stilbenoid	N/A	N/A	(2) Ptenf/f mice Pten+/f mice	(2) 10 mg/kg/3–30 weeks	(2) C-caspase3, p21, p27, Ac-p53/p53 ↑	[67]

N/A, not available; ↑, up-regulated; ↓, down-regulated.

**Table 4:** Dietary products regulating main apoptosis signal pathways.

Mechanism	Compounds
PARP	Artepillin C
	Auriculasin
	Delphinidin
	Dioscin
	Diketopiperazine disulfide glionitrin A
	Docetaxel
	Jungermannenone A and B
	LLDT-288
	Malformin A1
	Panduratin A
	Physlain A, B
	Tetrandrine
	Triterpenoid plectranthoic acid
	Viriditoxin
	Xanthohumol
	6α-acetoxyanopterine
Caspase	Artepillin C
	Delphinidin
	Dioscin
	Diketopiperazine disulfide glionitrin A
	FD1a, FD1c, FD1h, FD2a, FD2c, FD2h
	Flavonoid
	Guttiferone F
	Heteronemin
	Scutellarin
	Tetrandrine
	Thalicthuberine
	Triterpenoid plectranthoic acid
	Xanthohumol
	3-butenyl isothiocyanate
C-FLIP	Nexrutine
FADD	Panduratin A
RIPK1	Ophiopogonin D’
ROS	Auriculasin
HDAC	Delphinidin
ATF2	Solanine
Caspase-8	Delphinidin
p53	Pterostilbene
Beclin 1	Docetaxel
JNK	Docetaxel
Cytochrome C	Viriditoxin
Puma, Noxa	Delphinidin
V-ATPase inhibition	Bovine Milk Lactoferrin
Acinus	Panduratin A
Bcl-xL	Triterpenoid plectranthoic acid
Bim	Ophiopogonin D’
Akt	Tetrandrine
NF-kB	Chalcone
	Cardamonin
Bid	Diketopiperazine disulfide glionitrin A
	Ophiopogonin D’
	Panduratin A
	Ophiopogonin D’
Bcl-2	Auriculasin
	Dioscin
	Flavonoid
	Guttiferone F
	LLDT-288
	Malformin A1
	Panduratin A
	Scutellarin
	Solanine
	Tetrandrine
	Triterpenoid plectranthoic acid
	Viriditoxin
Bax	Auriculasin
	Delphinidin
	Dioscin
	Diketopiperazine disulfide glionitrin A
	FD1a, FD1c, FD1h, FD2a, FD2c, FD2h
	Flavonoid
	Guttiferone F
	LLDT-288

**Table 5:** Anti-angiogenesis inducing dietary compounds.

Compound	Classification	Source	Organism	Cell Line/Animal Model	Dose; Duration	Mechanism	Reference
Fucoidan	Polysaccharide	Algae	Protist	DU-145 xenografts	20 mg/kg/28 days	VEGF, Cyclin D1, Bcl-xL ↓	[68]
Nordihydroguaiaretic acid	Tetrol	*Larrea tridentate*	Plant	PC3	20 μM/0, 0.5, 1, 2, 3 h	NRP1 ↓	[69]
Terrestrosin D	Steroids	*Tribulus terrestris* L.	Plant	PC3, HUVEC	50 mg/kg/28 days		[49]
Resveratrol, Pterostilbene	Polyphenol,	Red wine	Plant	LNCaP, Du145 PC3 xenografts	5–100 µM/24 h	MTA 1↓	[70]
	Stilbenol				50 mg/kg/day/8 days		
Sesamin	Lignans	*Sesamum indicum*	Plant	DU145	100 μM/12 h	ICAM-1, MMP-9, VEGF↓	[75]
(1) Epigallocatechin-3-gallate	Polyphenol, Flavan	Green tea	Plant	CWR22R	(1) 50 mg/kg/20 days	(1), (2) CD31-positive endothelial cells ↓	[72]
(2) peracetate of EGCG					(2) 86.7 mg/kg/20 days		
Betulinic acid	Triterpenoid,	Birch bark	Plant	LNCaP/LNCaP xenografts	5, 10, 15, 20 μmol/L/24 h	VEGF, Sp proteins↓	[73]
	Hydroxy- monocarboxylic acid				10, 20 mg/kg/day/7 days		
(−)-Gossypol	N/A	Cottonseed	Plant	NCr-nu/nu nude mice injected with PC-3 cells	10 mg/kg × 5/day/4 weeks	Bcl-2 ↓	[74]

N/A, not available; ↓, down-regulated.

**Table 6:** Anti-metastasis inducing dietary compounds.

Compound	Classification	Source	Organism	Cell Line/Animal Model	Dose; Duration	Mechanism	Reference
Decursin, decursinol angelate	Coumarins	*Angelica gigas* Nakai	Plant	(1) C57BL/6 (2) TRAMP mice	(1) 5 mg/mouse/8, 16, 28 weeks (2) 3 mg/mouse/8, 16, 28 weeks	(1) Snail2, Twist, Notch1, TGFBR2, E-cadherin ↓ (2) Snail2 ↓	[76]
Luteolin, ellagic acid and punicic acid	Flavones,	Pomegranate	Plant	PCa xenograft SCID mice	64 µg/5 days/8 weeks	PLA2, COX, CXCR4, Gα_13_, PI3K, p-AKT ↓	[77]
	Polyphenol						

N/A, not available; ↓, down-regulated.

**Table 7:** MicroRNA (miRNA) regulating natural compounds.

Compound	Classification	Source	Organism	Cell Line/Animal Model	Dose; Duration	Mechanism	Reference
Luteolin, Ellagic acid, Punicic acid	Flavones, polyphenols	Pomegranate juice	Plant	PC3	8 µg/mL/12 h	miR144, miR-133b, miR-1, miR-122, miR-34c, miR-200c, miR-127, miR-335, miR-124, miR-181a, miR-7, miR-215, miR-15a, Let-7d, miR-20a, miR-21, miR-9, miR-29b, miR-181b↓	[79]
Epigallocatechin-3-gallate	Polyphenols, Flavan	Green tea	Plant	PCa xenografts	3 mg/6 weeks	miRNA-330↑ miRNA-21↓	[80]
Green tea polysaccharide	Polysaccharide	*Camellia sinensis* L.	Plant	PC3	25, 50, 100 μg/mL/48 h	miR-93↓	[81]
Resveratrol	Stilbenoid	N/A	Plant	DU145, LNCaP	50 mM/24 h	miR-17, miR-20a, miR-20b, miR-106a, miR-106b↑	[82]
Genistein	Isoflavone	N/A	Plant	PC3, DU145	25μM/4 days	sFRP1, Smad4↑ miR-1260b↓	[83]
Curcumin	Polyphenol	*Curcoma longa*	Plant	TRAMP C1	2.5 μM; 5 days	Nrf 2, NQO-1↑	[84]
EF24	Polyphenol	*Curcoma longa*	Plant	DU145	5 µM; 24 h	miR-345, miR-409, miR-10a miR-206↑ miR-21, miR-26a, miR-24, miR-30b miR-29a↓	[85]
Genistein, Daidzein	Isoflavones	Soy	Plant	PC-3, DU-145, LNCaP	40 μM, 110 μM; 48 h	GSTP1, EPHB2↑	[86]
Gefitinib, Luteolin	Anilinoquinazoline, Flavones	*Reseda luteola*	Plant	PC-3, U2OS	60 μM, 60 μM; 24 h, 48 h, 72 h	miR-630, miR-5703↑	[87]

N/A, not available; ↑, up-regulated; ↓, down-regulated.

**Table 8:** Multi-drug resistance sensitizing dietary compounds.

Compound	Classification	Source	Organism	Cell Line/Animal Model	Dose; Duration	Mechanism	Reference
6-gingerol, 10-gingerol, 6-shogaol, and 10-shogaol	Ketone, guaiacol	Ginger	Plant	PC3R	100 μM/24 h	MRP1, GSTπ↓	[33]
Scutellarin	Glycosyloxyflavone	*Scutellaria altissima* L.	Plant	PC3	200 µM/48 h	γ-H2AX foci ↑	[47]
	(Flavone)						
(8R)-3β,8-dihydroxypolypoda-13E,17E,21-triene	Bicyclic triterpenoid	*Pistacia lentiscus*	Plant	Drug-resistant PC-3 PC-3 tumor xenografts grown on the CAM	30 μM/48, 72 h	Z-VAD-FMK ↑	[88]
					10, 30, 100 μM/4 days		
(1) Theaflavins, (2) epicatechin-3-gallate, (3) epigallocatechin-3-gallate	Polyphenols	Black and green tea	Plant	LNCaP	(1) 100 μg/mL/24 h	MDR-1 ↓	[89]
					(2), (3) 1, 10 μM/24 h		

N/A, not available; ↑, up-regulated; ↓, down-regulated.

## Abbreviations

miRNA	microRNA
MDR	multi-drug-resistance
PARP	poly adenosine diphosphate ribose polymerase
PTEN	phosphatase and tensin homolog
Akt	protein kinase B
SHH	sonic hedgehog
FLIP	cellular FLICE (FADD-like IL-1β-converting enzyme)-inhibitory protein
VEGF	vascular endothelial growth factor
GM-CSF	granulocyte-macrophage colony-stimulating factor
IL-12	interleukin 12; KAI1, Kangai 1
OPG	osteoprotegerin; DNA, deoxyribonucleic Acid
BAX	BCL2 Associated X
LNCaP	lymph node carcinoma of the prostate
ROS	reactive oxygen species
NF-kB1	nuclear factor kappa-light-chain-enhancer of activated B cells 1
HDAC3	histone deacetylase 3
Puma	p53 upregulated modulator of apoptosis
NOXA	NADPH oxidase activator
JNK	Janus kinase
MMP	mitochondrial membrane potential
RIPK1	receptor-interacting serine/threonine-protein kinase 1
Bcl-2	B-cell lymphoma 2
Bim	Bcl*-2-*like protein 11
FADD	Fas-associated protein with death domain
TRAIL	TNF-related apoptosis inducing ligand
BITC	3-butenyl isothiocyanate
IC50	the half maximal inhibitory concentration
NK-kB	nuclear factor kappa light chain enhancer of activated B cells
MTA1	metastasis-associated protein 1
STAT3 IHC	signal transducer and activator of transcription 3 Immunohistochemistry
LLDT-288	(14S)-14β-(1-(2-morpholinoethyl)-1H-indazol-5-ylamino)mthylepitriptolide
ECM	extra-cellular matrix; EMT, epithelial-to-mesenchymal transition
TRAMP	transgenic adenocarcinoma of mouse prostate
NE-Ca	N-ethylcarboxamideadenosine
PLA2	phospholipases A2; COX, cyclooxygenase
AA	acetic acid
CXCR4	C-X-C chemokine receptor type 4
PI3K	phosphoinositide 3-kinases
CXCL12,	C-X-C motif chemokine ligand 12
MRP1	multidrug resistance-associated protein 1
GSTπ	glutathione S-transferase Pi
GST	glutathione S-transferase
PPV	personalized peptide vaccination
Mo-MDSC	monocytic myeloid-derived suppressor cells

## References

[1] Bray F., Ferlay J., Soerjomataram I., Siegel R.L., Torre L.A., Jemal A. (2018). Global cancer statistics 2018: Globocan estimates of incidence and mortality worldwide for 36 cancers in 185 countries. CA.

[2] Hauner K., Maisch P., Retz M. (2017). Side effects of chemotherapy. Der Urologe. Ausg. A.

[3] American Cancer Society Key Statistics for Prostate Cancer. https://www.cancer.org/cancer/prostate-cancer/about/key-statistics.html#references.

[4] Dogan S.E., Mizrak D., Alkan A., Demirkazik A. (2017). Docetaxel-induced pericardial effusion. J. Oncol. Pharm. Pract..

[5] Hughes C.L., Dhiman T.R. (2002). Dietary compounds in relation to dietary diversity and human health. J. Med. Food.

[6] Sung S., Kwon D., Um E., Kim B. (2019). Could polyphenols help in the control of rheumatoid arthritis?. Molecules.

[7] Direito R., Rocha J. (2019). Anti-inflammatory effects of persimmon (diospyros kaki l.) in experimental rodent rheumatoid arthritis. J. Diet. Suppl..

[8] Nwanna E.E., Ibukun E.O., Oboh G. (2019). Eggplant (solanum spp) supplemented fruits diet modulated the activities of ectonucleoside triphosphate diphosphohydrolase (entpdase), monoamine oxidase (mao), and cholinesterases (ache/bche) in the brain of diabetic wistar male rats. J. Food Biochem..

[9] Lee J.E., Song H.S., Park M.N., Kim S.H. (2018). Ethanol extract of oldenlandia diffusa herba attenuates scopolamine-induced cognitive impairments in mice via activation of bdnf, p-creb and inhibition of acetylcholinesterase. Int. J. Mol. Sci..

[10] Hwang D., Kim M., Park H., Jeong M.I., Jung W., Kim B. (2019). Natural products and acute myeloid leukemia: A review highlighting mechanisms of action. Nutrients.

[11] Abutaha N., Nasr F.A., Al-Zharani M., Alqahtani A.S. (2019). Effects of hexane root extract of ferula hermonis boiss. On human breast and colon cancer cells: An in vitro and in vivo study. BioMed Res. Int..

[12] Noman O.M., Mubarak M., Abdelhabib S., Wadaan M.A., Shen H., Qu Z., Harata-Lee Y., Aung T.N., Cui J., Wang W. (2019). Understanding the mechanistic contribution of herbal extracts in compound kushen injection with transcriptome analysis. BioMed Res. Int..

[13] Kim C., Kim B. (2018). Anti-cancer natural products and their bioactive compounds inducing er stress-mediated apoptosis: A review. Nutrients.

[14] Asif M. (2011). Health effects of omega-3,6,9 fatty acids: Perilla frutescens is a good example of plant oils. Orient. Pharm. Exp. Med..

[15] Nobili S., Lippi D., Witort E., Donnini M., Bausi L., Mini E., Capaccioli S. (2009). Natural compounds for cancer treatment and prevention. Pharmacol. Res..

[16] Banikazemi Z., Haji H.A., Mohammadi M., Taheripak G., Iranifar E., Poursadeghiyan M., Moridikia A., Rashidi B., Taghizadeh M., Mirzaei H. (2018). Diet and cancer prevention: Dietary compounds, dietary micrornas, and dietary exosomes. J. Cell Biochem..

[17] Qiang Z., Meng L., Yi C., Yu L., Chen W., Sha W. (2019). Curcumin regulates the mir-21/pten/akt pathway and acts in synergy with pd98059 to induce apoptosis of human gastric cancer mgc-803 cells. J. Int. Med. Res..

[18] Feng T., Wei Y., Lee R.J., Zhao L. (2017). Liposomal curcumin and its application in cancer. Int. J. Nanomed..

[19] Fu J., Shrivastava A., Shrivastava S.K., Srivastava R.K., Shankar S. (2019). Triacetyl resveratrol upregulates mirna200 and suppresses the shh pathway in pancreatic cancer: A potential therapeutic agent. Int. J. Oncol..

[20] Ko J.-H., Sethi G., Um J.-Y., Shanmugam M.K., Arfuso F., Kumar A.P., Bishayee A., Ahn K.S. (2017). The role of resveratrol in cancer therapy. Int. J. Mol. Sci..

[21] Gao Z., Gao W., Zeng S.-L., Li P., Liu E.H. (2018). Chemical Structures, Bioactivities and Molecular Mechanisms of Citrus Polymethoxyflavones. J. Funct. Foods..

[22] Hassan M., Watari H., AbuAlmaaty A., Ohba Y., Sakuragi N. (2014). Apoptosis and molecular targeting therapy in cancer. BioMed Res. Int..

[23] Prager G.W., Poettler M., Unseld M., Zielinski C.C. (2012). Angiogenesis in cancer: Anti-vegf escape mechanisms. Transl. Lung Cancer Res..

[24] Park G., Choi K.-C. (2016). Advanced New Strategies for Metastatic Cancer Treatment by Therapeutic Stem Cells and Oncolytic Virotherapy. Oncotarget.

[25] Cornelison R., Llaneza C.D., Landen N.C. (2017). Emerging therapeutics to overcome chemoresistance in epithelial ovarian cancer: A mini-review. Int. J. Mol. Sci..

[26] Adiwidjaja J., McLachlan A.J., Boddy A.V. (2017). Curcumin as a clinically-promising anti-cancer agent: Pharmacokinetics and drug interactions. Expert Opin. Drug Metab. Toxicol..

[27] Cho H.D., Lee J.H., Moon K.D., Park K.H., Lee M.K., Seo K.I. (2018). Auriculasin-induced ros causes prostate cancer cell death via induction of apoptosis. Food Chem. Toxicol..

[28] Pascoal A.C., Ehrenfried C.A., Lopez B.G., de Araujo T.M., Pascoal V.D., Gilioli R., Anhe G.F., Ruiz A.L., Carvalho J.E., Stefanello M.E. (2014). Antiproliferative activity and induction of apoptosis in pc-3 cells by the chalcone cardamonin from campomanesia adamantium (myrtaceae) in a bioactivity-guided study. Molecules.

[29] Deb Majumdar I., Devanabanda A., Fox B., Schwartzman J., Cong H., Porco J.A., Weber H.C. (2011). Synthetic cyclohexenyl chalcone natural products possess cytotoxic activities against prostate cancer cells and inhibit cysteine cathepsins in vitro. Biochem. Biophys. Res. Commun..

[30] Jeong M.H., Ko H., Jeon H., Sung G.J., Park S.Y., Jun W.J., Lee Y.H., Lee J., Lee S.W., Yoon H.G. (2016). Delphinidin induces apoptosis via cleaved hdac3-mediated p53 acetylation and oligomerization in prostate cancer cells. Oncotarget.

[31] Park K.H., Yin J., Yoon K.H., Hwang Y.J., Lee M.W. (2016). Antiproliferative effects of new dimeric ellagitannin from cornus alba in prostate cancer cells including apoptosis-related s-phase arrest. Molecules.

[32] Puente J., Grande E., Medina A., Maroto P., Lainez N., Arranz J.A. (2017). Docetaxel in prostate cancer: A familiar face as the new standard in a hormone-sensitive setting. Ther. Adv. Med. Oncol..

[33] Wang Q., He W.Y., Zeng Y.Z., Hossain A., Gou X. (2018). Inhibiting autophagy overcomes docetaxel resistance in castration-resistant prostate cancer cells. Int. Urol. Nephrol..

[34] Zheng L., Chen S., Cao Y., Zhao L., Gao Y., Ding X., Wang X., Gu Y., Wang S., Zhu Z. (2018). Combination of comprehensive two-dimensional prostate cancer cell membrane chromatographic system and network pharmacology for characterizing membrane binding active components from radix et rhizoma rhei and their targets. J. Chromatogr. A.

[35] Hanafi M.M.M., Afzan A., Yaakob H., Aziz R., Sarmidi M.R., Wolfender J.L., Prieto J.M. (2017). In vitro pro-apoptotic and anti-migratory effects of ficus deltoidea l. Plant extracts on the human prostate cancer cell lines pc3. Front. Pharmacol..

[36] Ding Y., Ren K., Dong H., Song F., Chen J., Guo Y., Liu Y., Tao W., Zhang Y. (2017). Flavonoids from persimmon (diospyros kaki l.) leaves inhibit proliferation and induce apoptosis in pc-3 cells by activation of oxidative stress and mitochondrial apoptosis. Chem. Biol. Interact..

[37] Li X., Lao Y., Zhang H., Wang X., Tan H., Lin Z., Xu H. (2015). The natural compound guttiferone f sensitizes prostate cancer to starvation induced apoptosis via calcium and jnk elevation. BMC Cancer.

[38] Guo Y.X., Lin Z.M., Wang M.J., Dong Y.W., Niu H.M., Young C.Y., Lou H.X., Yuan H.Q. (2016). Jungermannenone a and b induce ros- and cell cycle-dependent apoptosis in prostate cancer cells in vitro. Acta Pharmacol. Sin..

[39] Zhao Y., Chen R., Wang Y., Qing C., Wang W., Yang Y. (2017). In vitro and in vivo efficacy studies of lavender angustifolia essential oil and its active constituents on the proliferation of human prostate cancer. Integr. Cancer Ther..

[40] Wang Q., Xu J., Li X., Zhang D., Han Y., Zhang X. (2017). Comprehensive two-dimensional pc-3 prostate cancer cell membrane chromatography for screening anti-tumor components from radix sophorae flavescentis. J. Sep. Sci..

[41] Zhang Y., Li L., Wang J., Cheng W., Zhang J., Li X., Zhang Z., Gong J., Ghosh R., Kumar A.P. (2017). Combination of nexrutine and docetaxel suppresses nfkappab-mediated activation of c-flip. Mol. Carcinog..

[42] Lu Z., Wang H., Zhu M., Song W., Wang J., Wu C., Kong Y., Guo J., Li N., Liu J. (2018). Ophiopogonin D′, a natural product from radix ophiopogonis, induces in vitro and in vivo ripk1-dependent and caspase-independent apoptotic death in androgen-independent human prostate cancer cells. Front. Pharmacol..

[43] Yun J.M., Kweon M.H., Kwon H., Hwang J.K., Mukhtar H. (2006). Induction of apoptosis and cell cycle arrest by a chalcone panduratin a isolated from kaempferia pandurata in androgen-independent human prostate cancer cells pc3 and du145. Carcinogenesis.

[44] Adaramoye O., Erguen B., Nitzsche B., Hopfner M., Jung K., Rabien A. (2017). Punicalagin, a polyphenol from pomegranate fruit, induces growth inhibition and apoptosis in human pc-3 and lncap cells. Chem. Biol. Interact..

[45] Han H., Qiu L., Wang X., Qiu F., Wong Y., Yao X. (2011). Physalins a and b inhibit androgen-independent prostate cancer cell growth through activation of cell apoptosis and downregulation of androgen receptor expression. Biol. Pharm. Bull..

[46] Amujuri D., Siva B., Poornima B., Sirisha K., Sarma A.V.S., Lakshma Nayak V., Tiwari A.K., Purushotham U., Suresh Babu K. (2018). Synthesis and biological evaluation of schizandrin derivatives as potential anti-cancer agents. Eur. J. Med. Chem..

[47] Gao C., Zhou Y., Jiang Z., Zhao Y., Zhang D., Cong X., Cao R., Li H., Tian W. (2017). Cytotoxic and chemosensitization effects of scutellarin from traditional chinese herb scutellaria altissima l. In human prostate cancer cells. Oncol. Rep..

[48] Pan B., Zhong W., Deng Z., Lai C., Chu J., Jiao G., Liu J., Zhou Q. (2016). Inhibition of prostate cancer growth by solanine requires the suppression of cell cycle proteins and the activation of ros/p38 signaling pathway. Cancer Med..

[49] Wei S., Fukuhara H., Chen G., Kawada C., Kurabayashi A., Furihata M., Inoue K., Shuin T. (2014). Terrestrosin d, a steroidal saponin from tribulus terrestris l., inhibits growth and angiogenesis of human prostate cancer in vitro and in vivo. Pathobiology.

[50] Liu W., Kou B., Ma Z.K., Tang X.S., Lv C., Ye M., Chen J.Q., Li L., Wang X.Y., He D.L. (2015). Tetrandrine suppresses proliferation, induces apoptosis, and inhibits migration and invasion in human prostate cancer cells. Asian J. Androl..

[51] Levrier C., Rockstroh A., Gabrielli B., Kavallaris M., Lehman M., Davis R.A., Sadowski M.C., Nelson C.C. (2018). Discovery of thalicthuberine as a novel antimitotic agent from nature that disrupts microtubule dynamics and induces apoptosis in prostate cancer cells. Cell Cycle.

[52] Akhtar N., Syed D.N., Khan M.I., Adhami V.M., Mirza B., Mukhtar H. (2016). The pentacyclic triterpenoid, plectranthoic acid, a novel activator of ampk induces apoptotic death in prostate cancer cells. Oncotarget.

[53] Klosek M., Mertas A., Krol W., Jaworska D., Szymszal J., Szliszka E. (2016). Tumor necrosis factor-related apoptosis-inducing ligand-induced apoptosis in prostate cancer cells after treatment with xanthohumol-a natural compound present in humulus lupulus l. Int. J. Mol. Sci..

[54] Arora R., Kumar R., Mahajan J., Vig A.P., Singh B., Singh B., Arora S. (2016). 3-butenyl isothiocyanate: A hydrolytic product of glucosinolate as a potential cytotoxic agent against human cancer cell lines. J. Food Sci. Technol..

[55] Levrier C., Sadowski M.C., Rockstroh A., Gabrielli B., Kavallaris M., Lehman M., Davis R.A., Nelson C.C. (2017). 6alpha-acetoxyanopterine: A novel structure class of mitotic inhibitor disrupting microtubule dynamics in prostate cancer cells. Mol. Cancer Ther..

[56] Endo S., Hoshi M., Matsunaga T., Inoue T., Ichihara K., Ikari A. (2018). Autophagy inhibition enhances anticancer efficacy of artepillin c, a cinnamic acid derivative in brazilian green propolis. Biochem. Biophys. Res. Commun..

[57] Kim Y.J., Park H.B., Yoo J.H., Kwon H.C., Kim J., Yang H.O. (2014). Glionitrin a, a new diketopiperazine disulfide, activates atm-atr-chk1/2 via 53bp1 phosphorylation in du145 cells and shows antitumor effect in xenograft model. Biol. Pharm. Bull..

[58] Mukhopadhyay A., Hanold L.E., Thayele Purayil H., Gisemba S.A., Senadheera S.N., Aldrich J.V. (2017). Macrocyclic peptides decrease c-myc protein levels and reduce prostate cancer cell growth. Cancer Biol. Ther..

[59] Liu Y., Wang M., Wang D., Li X., Wang W., Lou H., Yuan H. (2016). Malformin a1 promotes cell death through induction of apoptosis, necrosis and autophagy in prostate cancer cells. Cancer Chemother. Pharmacol..

[60] Kundu S., Kim T.H., Yoon J.H., Shin H.S., Lee J., Jung J.H., Kim H.S. (2014). Viriditoxin regulates apoptosis and autophagy via mitotic catastrophe and microtubule formation in human prostate cancer cells. Int. J. Oncol..

[61] Wu S.Y., Sung P.J., Chang Y.L., Pan S.L., Teng C.M. (2015). Heteronemin, a spongean sesterterpene, induces cell apoptosis and autophagy in human renal carcinoma cells. BioMed Res. Int..

[62] Lee M.G., Liu Y.C., Lee Y.L., El-Shazly M., Lai K.H., Shih S.P., Ke S.C., Hong M.C., Du Y.C., Yang J.C. (2018). Heteronemin, a marine sesterterpenoid-type metabolite, induces apoptosis in prostate lncap cells via oxidative and er stress combined with the inhibition of topoisomerase ii and hsp90. Mar. Drugs.

[63] Guedes J.P., Pereira C.S., Rodrigues L.R., Corte-Real M. (2018). Bovine milk lactoferrin selectively kills highly metastatic prostate cancer pc-3 and osteosarcoma mg-63 cells in vitro. Front. Oncol..

[64] Sato C., Kaneko S., Sato A., Virgona N., Namiki K., Yano T. (2017). Combination effect of delta-tocotrienol and gamma-tocopherol on prostate cancer cell growth. J. Nutr. Sci. Vitaminol..

[65] Tao X., Xu L., Yin L., Han X., Qi Y., Xu Y., Song S., Zhao Y., Peng J. (2017). Dioscin induces prostate cancer cell apoptosis through activation of estrogen receptor-beta. Cell Death Dis..

[66] Xu H., Fan X., Zhang G., Liu X., Li Z., Li Y., Jiang B. (2017). Lldt-288, a novel triptolide analogue exhibits potent antitumor activity in vitro and in vivo. Biomed. Pharmacother. Biomed. Pharmacother..

[67] Dhar S., Kumar A., Zhang L., Rimando A.M., Lage J.M., Lewin J.R., Atfi A., Zhang X., Levenson A.S. (2016). Dietary pterostilbene is a novel mta1-targeted chemopreventive and therapeutic agent in prostate cancer. Oncotarget.

[68] Rui X., Pan H.F., Shao S.L., Xu X.M. (2017). Anti-tumor and anti-angiogenic effects of fucoidan on prostate cancer: Possible jak-stat3 pathway. BMC Complement Alt. Med..

[69] Li X., Fan S., Pan X., Xiaokaiti Y., Duan J., Shi Y., Pan Y., Tie L., Wang X., Li Y. (2016). Nordihydroguaiaretic acid impairs prostate cancer cell migration and tumor metastasis by suppressing neuropilin 1. Oncotarget.

[70] Li K., Dias S.J., Rimando A.M., Dhar S., Mizuno C.S., Penman A.D., Lewin J.R., Levenson A.S. (2013). Pterostilbene acts through metastasis-associated protein 1 to inhibit tumor growth, progression and metastasis in prostate cancer. PLoS ONE.

[71] Ryu N.H., Park K.R., Kim S.M., Yun H.M., Nam D., Lee S.G., Jang H.J., Ahn K.S., Kim S.H., Shim B.S. (2012). A hexane fraction of guava leaves (psidium guajava l.) induces anticancer activity by suppressing akt/mammalian target of rapamycin/ribosomal p70 s6 kinase in human prostate cancer cells. J. Med. Food.

[72] Lee S.C., Chan W.K., Lee T.W., Lam W.H., Wang X., Chan T.H., Wong Y.C. (2008). Effect of a prodrug of the green tea polyphenol (-)-epigallocatechin-3-gallate on the growth of androgen-independent prostate cancer in vivo. Nutr. Cancer.

[73] Chintharlapalli S., Papineni S., Lei P., Pathi S., Safe S. (2011). Betulinic acid inhibits colon cancer cell and tumor growth and induces proteasome-dependent and -independent downregulation of specificity proteins (sp) transcription factors. BMC Cancer.

[74] Xu L., Yang D., Wang S., Tang W., Liu M., Davis M., Chen J., Rae J.M., Lawrence T., Lippman M.E. (2005). (-)-gossypol enhances response to radiation therapy and results in tumor regression of human prostate cancer. Mol. Cancer Ther..

[75] Harikumar K.B., Sung B., Tharakan S.T., Pandey M.K., Joy B., Guha S., Krishnan S., Aggarwal B.B. (2010). Sesamin manifests chemopreventive effects through the suppression of nf-kappa b-regulated cell survival, proliferation, invasion, and angiogenic gene products. Mol. Cancer Res. MCR.

[76] Tang S.N., Zhang J., Wu W., Jiang P., Puppala M., Zhang Y., Xing C., Kim S.H., Jiang C., Lü J. (2015). Chemopreventive effects of korean angelica versus its major pyranocoumarins on two lineages of transgenic adenocarcinoma of mouse prostate carcinogenesis. Cancer Prev. Res..

[77] Wang L., Li W., Lin M., Garcia M., Mulholland D., Lilly M., Martins-Green M. (2014). Luteolin, ellagic acid and punicic acid are natural products that inhibit prostate cancer metastasis. Carcinogenesis.

[78] Mehta R.G., Murillo G., Naithani R., Peng X. (2010). Cancer chemoprevention by natural products: How far have we come?. Pharm. Res..

[79] Wang L., Ho J., Glackin C., Martins-Green M. (2012). Specific pomegranate juice components as potential inhibitors of prostate cancer metastasis. Transl. Oncol..

[80] Siddiqui I.A., Asim M., Hafeez B.B., Adhami V.M., Tarapore R.S., Mukhtar H. (2011). Green tea polyphenol egcg blunts androgen receptor function in prostate cancer. FASEB J..

[81] Yang K., Gao Z.Y., Li T.Q., Song W., Xiao W., Zheng J., Chen H., Chen G.H., Zou H.Y. (2019). Anti-tumor activity and the mechanism of a green tea (camellia sinensis) polysaccharide on prostate cancer. Int. J. Biol. Macromol..

[82] Dhar S., Hicks C., Levenson A.S. (2011). Resveratrol and prostate cancer: Promising role for micrornas. Mol. Nutr. Food Res..

[83] Hirata H., Hinoda Y., Shahryari V., Deng G., Tanaka Y., Tabatabai Z.L., Dahiya R. (2014). Genistein downregulates onco-mir-1260b and upregulates sfrp1 and smad4 via demethylation and histone modification in prostate cancer cells. Br. J. Cancer.

[84] Khor T.O., Huang Y., Wu T.Y., Shu L., Lee J., Kong A.N. (2011). Pharmacodynamics of curcumin as DNA hypomethylation agent in restoring the expression of nrf2 via promoter cpgs demethylation. Biochem. Pharmacol..

[85] Yang C.H., Yue J., Sims M., Pfeffer L.M. (2013). The curcumin analog ef24 targets nf-κb and mirna-21, and has potent anticancer activity in vitro and in vivo. PLoS ONE.

[86] Vardi A., Bosviel R., Rabiau N., Adjakly M., Satih S., Dechelotte P., Boiteux J.P., Fontana L., Bignon Y.J., Guy L. (2010). Soy phytoestrogens modify DNA methylation of gstp1, rassf1a, eph2 and brca1 promoter in prostate cancer cells. In Vivo.

[87] Sakurai M.A., Ozaki Y., Okuzaki D., Naito Y., Sasakura T., Okamoto A., Tabara H., Inoue T., Hagiyama M., Ito A. (2014). Gefitinib and luteolin cause growth arrest of human prostate cancer pc-3 cells via inhibition of cyclin g-associated kinase and induction of mir-630. PLoS ONE.

[88] Morad S.A., Schmidt C., Buchele B., Schneider B., Wenzler M., Syrovets T., Simmet T. (2011). (8r)-3beta,8-dihydroxypolypoda-13e,17e,21-triene induces cell cycle arrest and apoptosis in treatment-resistant prostate cancer cells. J. Nat. Prod..

[89] Lyn-Cook B.D., Rogers T., Yan Y., Blann E.B., Kadlubar F.F., Hammons G.J. (1999). Chemopreventive effects of tea extracts and various components on human pancreatic and prostate tumor cells in vitro. Nutr. Cancer.

[90] Steentjes L., Siesling S., Drummond F.J., van Manen J.G., Sharp L., Gavin A. (2018). Factors associated with current and severe physical side-effects after prostate cancer treatment: What men report. Eur. J. Cancer Care.

[91] Wang Y., Shi J., Chai K., Ying X., Zhou B.P. (2013). The role of snail in emt and tumorigenesis. Curr. Cancer Drug Targ..

[92] Geiger T.R., Peeper D.S. (2009). Metastasis mechanisms. Biochim. Biophys. Acta.

[93] Jain C.K., Gupta A., Dogra N., Kumar V.S., Wadhwa G., Sharma S.K. (2014). Microrna therapeutics: The emerging anticancer strategies. Recent Pat. Anticancer Drug Discov..

[94] Brase J.C., Johannes M., Schlomm T., Fälth M., Haese A., Steuber T., Beissbarth T., Kuner R., Sültmann H. (2011). Circulating mirnas are correlated with tumor progression in prostate cancer. Int. J. Cancer.

[95] Porkka K.P., Pfeiffer M.J., Waltering K.K., Vessella R.L., Tammela T.L.J., Visakorpi T. (2007). Microrna expression profiling in prostate cancer. Cancer Res..

[96] Peng Y., Croce C.M. (2016). The role of micrornas in human cancer. Signal Transduct. Targ. Ther..

[97] Krasnov G.S., Dmitriev A.A., Sadritdinova A.F., Volchenko N.N., Slavnova E.N., Danilova T.V., Snezhkina A.V., Melnikova N.V., Fedorova M.S., Lakunina V.A. (2015). Molecular genetic mechanisms of drug resistance in prostate cancer. Mol. Biol..

[98] Kumar A., Jaitak V. (2019). Natural products as multidrug resistance modulators in cancer. Eur. J. Med. Chem..

[99] Guo Q., Cao H., Qi X., Li H., Ye P., Wang Z., Wang D., Sun M. (2017). Research progress in reversal of tumor multi-drug resistance via natural products. Anticancer Agents Med. Chem..

[100] Baguley B.C. (2010). Multiple drug resistance mechanisms in cancer. Mol. Biotechnol..

[101] Langer R., Ott K., Feith M., Lordick F., Specht K., Becker K., Hofler H. (2010). High pretherapeutic thymidylate synthetase and mrp-1 protein levels are associated with nonresponse to neoadjuvant chemotherapy in oesophageal adenocarcinoma patients. J. Surg. Oncol..

[102] Shi H., Lu D., Shu Y., Shi W., Lu S., Wang K. (2008). Expression of multidrug resistance-related proteins p-glycoprotein, glutathione-s-transferases, topoisomerase-ii and lung resistance protein in primary gastric cardiac adenocarcinoma. Hepatogastroenterology.

[103] Deb G., Shankar E., Thakur V.S., Ponsky L.E., Bodner D.R., Fu P., Gupta S. (2019). Green tea-induced epigenetic reactivation of tissue inhibitor of matrix metalloproteinase-3 suppresses prostate cancer progression through histone-modifying enzymes. Mol. Carcinog..

[104] Shankar E., Kanwal R., Candamo M., Gupta S. (2016). Dietary phytochemicals as epigenetic modifiers in cancer: Promise and challenges. Semin. Cancer Biol..

[105] Gathirua-Mwangi W.G., Zhang J. (2014). Dietary factors and risk for advanced prostate cancer. Eur. J. Cancer Prev..

[106] Canene-Adams K., Lindshield B.L., Wang S., Jeffery E.H., Clinton S.K., Erdman J.W. (2007). Combinations of tomato and broccoli enhance antitumor activity in dunning r3327-h prostate adenocarcinomas. Cancer Res..

[107] Taylor W.F., Moghadam S.E., Moridi Farimani M., Ebrahimi S.N., Tabefam M., Jabbarzadeh E. (2019). A multi-targeting natural compound with growth inhibitory and anti-angiogenic properties re-sensitizes chemotherapy resistant cancer. PLoS ONE.

[108] Imran M., Rauf A., Khan I.A., Shahbaz M., Qaisrani T.B., Fatmawati S., Abu-Izneid T., Imran A., Rahman K.U., Gondal T.A. (2018). Thymoquinone: A novel strategy to combat cancer: A review. Biomed. Pharmacother. Biomed. Pharmacother..

[109] Dybkowska E., Sadowska A., Swiderski F., Rakowska R., Wysocka K. (2018). The occurrence of resveratrol in foodstuffs and its potential for supporting cancer prevention and treatment. A review. Roczniki Panstwowego Zakladu Higieny.

[110] Shokoohinia Y., Jafari F., Mohammadi Z., Bazvandi L., Hosseinzadeh L., Chow N., Bhattacharyya P., Farzaei M.H., Farooqi A.A., Nabavi S.M. (2018). Potential anticancer properties of osthol: A comprehensive mechanistic review. Nutrients.

[111] Dahut W.L., Gulley J.L., Arlen P.M., Liu Y., Fedenko K.M., Steinberg S.M., Wright J.J., Parnes H., Chen C.C., Jones E. (2004). Randomized phase ii trial of docetaxel plus thalidomide in androgen-independent prostate cancer. J. Clin. Oncol..

[112] Koga N., Moriya F., Waki K., Yamada A., Itoh K., Noguchi M. (2017). Immunological efficacy of herbal medicines in prostate cancer patients treated by personalized peptide vaccine. Cancer Sci..

[113] Paller C.J., Denmeade S.R., Carducci M.A. (2016). Challenges of conducting clinical trials of natural products to combat cancer. Clin. Adv. Hematol. Oncol. H&O.

[114] Shankar E., Goel A., Gupta K., Gupta S. (2017). Plant flavone apigenin: An emerging anticancer agent. Curr. Pharmacol. Rep..

[115] Baker J., Ajani J., Scotté F., Winther D., Martin M., Aapro M.S., von Minckwitz G. (2009). Docetaxel-related side effects and their management. Eur. J. Oncol. Nurs..

